# Association of *IKZF1* and *CDKN2A* gene polymorphisms with childhood acute lymphoblastic leukemia: a high-resolution melting analysis

**DOI:** 10.1186/s12920-022-01325-6

**Published:** 2022-08-05

**Authors:** Mahla Sattarzadeh Bardsiri, Shahrzad Zehtab, Najibe Karami, Alireza Farsinejad, Mohsen Ehsan, Ahmad Fatemi

**Affiliations:** 1 Cell Therapy and Regenerative Medicine Comprehensive Center, Kerman University of Medical Sciences, Kerman, Iran; 2 Department of Hematology and Medical Laboratory Sciences, Faculty of Allied Medicine, Kerman University of Medical Sciences, Kerman, Iran; 3 Department of Hematology and Blood Banking, Faculty of Allied Medicine, Iran University of Medical Science, Tehran, Iran; 4 Cellular and Molecular Research Center, Gerash University of Medical Sciences, Gerash, Iran

**Keywords:** Childhood acute lymphoblastic leukemia, *IKZF1*, *CDKN2A*, Single nucleotide polymorphism, High-resolution melting analysis

## Abstract

**Background:**

Acute lymphoblastic leukemia is the most prevailing pediatric hematologic malignancy, and various factors such as environmental exposures and genetic variation affect ALL susceptibility and patients outcome. According to genome-wide association studies, several single nucleotide polymorphisms (SNPs) in *IKZF1* (rs4132601) and *CDKN2A* (rs3731249 and rs3731217) genes are associated with ALL susceptibility. Hereupon, this study aimed to discover the association between these SNPs and the risk of childhood ALL among a sample of the Iranian population.

**Methods:**

A total of fifty children with ALL were included in this case–control study, along with an additional fifty healthy children, matched for age and gender. High-resolution melting (HRM) analysis was employed to genotyping rs4132601, rs3731249, and rs3731217.

**Results:**

In the patient group, the CT genotype and T allele frequency of rs3731249 were significantly greater than controls (*p* = 0.01 and *p* = 0.005, respectively). Moreover, the positive association of CT and dominant model (CT + TT) genotypes and T allele at rs3731249 with the risk of ALL was confirmed (OR = 9.56, OR = 10.76 and OR = 11.00, respectively). There was no significant relation between rs4132601 (*IKZF1*), rs3731217 (*CDKN2A*), and childhood ALL.

**Conclusion:**

The present study indicates that CT genotype and T allele at rs3731249 (*CDKN2A*) can significantly increase the risk of ALL among children.

## Background

Acute lymphoblastic leukemia (ALL) is a heterogeneous disorder that arises from a malignant transformation and uncontrolled proliferation of lymphoid progenitors in peripheral blood, lymph nodes, bone marrow, and thymus [1, 2]. It is the most prevailing pediatric hematologic malignancy and chiefly targets those aged 2–5 years old; However, it has been shown that this malignancy can affect both children and middle-aged adults [3, 4]. Although the precise causes of ALL occurrence are yet to be determined, it is suggested that a combination of genetic susceptibility and environmental factors contribute to disease development [5, 6]. Recently, various genome-wide association studies (GWASs) have shown that inherited genetic variations can be beneficial markers for predicting ALL susceptibility, drug response, and risk of relapse in children [7, 8]. Single nucleotide polymorphisms (SNPs) are the most common type of germline variation (> 1% frequency) resulting from base-pair differences in genome sequences that are employed for studying genetic differences between populations. In the recent era, these valuable biological markers have been used for revealing the heritable risk of various diseases and are considered the major characters in disease-association studies in different races. The occurrence of SNPs in a gene or its regulatory region may disrupt its normal function [9–11]. There are three vital systems (xenobiotic, DNA repair, and cell regulation) that SNPs in genes involved in these pathways heighten the risk of acute leukemia [12]. Based on the GWASs, it is reported that common alterations in several genes are linked with B-cell homeostasis (*CEBPE*, *IKZF1*, *ARID5B*) and cell cycle regulation (*CDKN2A*) may affect children's susceptibility to ALL [13–15].

Ikaros family zinc finger 1 (*IKZF1;* 7p12.2) encodes a DNA-binding transcription factor with zinc finger motifs involved in hematopoiesis, particularly in the development and differentiation of lymphoid lineages [16–19]. Germline variation (SNPs) or mutations in the *IKZF1* gene or expression of a dominant-negative isoform of this gene (caused by deletion of zinc finger domains) can be related to several hematological malignancies [20, 21]. The association between several SNPs in the *IKZF1* gene (rs4132601, rs6964823, rs6944602, and rs11978267) and inherited susceptibility to ALL has been repeatedly declared in GWASs [22].

Cyclin-dependent kinase inhibitor 2A (*CDKN2A;* 9p21.3*)* encodes p16^INK4A^ and p14^ARF^, which are vital regulators of the cell cycle and play a significant role in programmed cell death through their tumor-suppressive activities [23, 24]. Thus, with deletion, mutation, or genetic polymorphisms of *CDKN2A,* the expression and effectiveness of p16^INK4A^ and p14^ARF^ tumor suppressors are decreased, leading to deregulation of cell proliferation and cancer development [25, 26]. Previous studies showed that *CDKN2A* genetic variations (rs3731217 and rs3731249) could be considered risk factors for both B-ALL and T-ALL [27].

Although numerous studies have been conducted concerning the link between *IKZF1* and *CDKN2A* genes and ALL pathogenesis, their findings are contradictory, probably due to the different clinical features, including ethnicity, age, and subtypes. Therefore, we selected the most prevalent SNPs of these genes (*IKZF1*; rs4132601 T > G, *CDKN2A*; rs3731217 A > C, and rs3731249 C > T) and evaluated them in the Iranian ALL population. Given the racially dependent distribution of these SNPs, evaluation of these variations in different populations seems to provide precious information about ALL susceptibility and prognosis. To achieve this objective, we designed an accurate and cost-effective high-resolution melting (HRM) analysis to assess the frequency of mentioned SNPs in the target population.

## Materials and methods

### Study subjects & sample collection

This case–control study included 50 ALL-diagnosed and 50 age- and sex-matched healthy children. The sample size estimation was conducted by the PGA software [28] with a two-sided alpha error of 5% and a power of 80%. In fact, all available samples were collected but we could not achieve the calculated sample size. According to the current World Health Organization (WHO) criteria, the disease was diagnosed and verified in the patient group. The healthy group comprised children with a normal complete blood count (CBC) and no history of malignancy or underlying disorders referred to the hospital for human leukocyte antigen (HLA) typing of bone marrow donation. By checking the identification documents of these children and their parents, and also based on the information collected from the design questionnaires, Iranian nationality was verified for both the case and control groups. The study gained the approval of the Kerman University of Medical Sciences ethical committee (ethics approval code: IR.KMU.REC.1397.074). All participant parents provided written informed permission before collecting study samples. Subsequently, genomic DNA was extracted from peripheral blood using a modified salting-out method [29]. Next, DNA samples were stored at − 20 °C until genotyping.

### HRM analysis for *IKZF1* and *CDKN2A* gene polymorphisms

#### Primer design

SNP database of NCBI (https://www.ncbi.nlm.nih.gov/snp/?term =) and Ensembl genome browser (http://asia.ensembl.org/index.html) were employed to provide the sequences of rs4132601 (*IKZF1*gene), rs3731217, and rs3731249 (*CDKN2A* gene). Furthermore, Primer3Plus (http://bioinformatics.nl/cgi-bin/primer3plus/primer3plus.cgi) and Oligo Analyzer Tool-Integrated DNA Technologies (http://eu.idtdna.com/calc/analyzer) were utilized to perform primer design and specificity analysis.

#### HRM technique

Genotyping of controls and patients was evaluated in a completely blinded fashion using the HRM technique and the Rotor-Gene® Q instrument (QIAGEN’s real-time PCR cycler) was used for this experiment. As the manufacturer stated in the protocol of 5 × HOT FIREPol® EvaGreen® HRM Mix (Solis BioDyne, Estonia), elements of an HRM reaction involved: 100 nM forward primer (10 pmol/ μL), 100 nM reverse primer (10 pmol/ μL), 1X HRM Master Mix, 50 ng/ μL DNA template and H2O PCR grade. It should be noted that the ultimate volume of the reaction mixture was 20μL. Later, DNA amplification occurred under these cycling circumstances: 12-min initial activation at 95 °C, followed by 40 amplification cycles comprised of 15-s denaturation at 95 °C, 20-s annealing at 67 °C, and extension at 72 °C for 20 s. Eventually, as the temperature increased from 65^˚^C to 90^˚^C at a ramp rate of 0.1 °C per second, an HRM step was performed. The collected data were investigated using the Rotor-Gene Q Series Software version 2.3.5.

### DNA sequencing

Because of obtaining different groups of melt curves, and to confirm the accuracy of genotyping by HRM assay, three samples from each group were submitted to BIONEER Corporation (South Korea) for direct DNA sequencing.

### Statistical analysis

To compare the case and control groups in terms of allele frequency and genotype distribution and to determine the predicted frequency of control genotypes, the Pearson Chi-square test and Hardy–Weinberg equilibrium were employed, respectively. In addition, multivariate logistic regression was applied to calculate odds ratios (ORs) and 95% confidence intervals (CIs) to assess an association between each genotype and the risk of ALL. Statistical analyses were conducted via SPSS software version 23.0, and a *p-value* less than 0.05 was regarded as statistically significant.

## Results

### Baseline characteristics of patients and controls

Our study included 50 children with ALL (age 5.5 ± 5.4 years) and 50 healthy children (age 6.2 ± 2.8 years). The demographic and hematologic characteristics of participants are presented in Table 1. All subjects in both case and control groups were matched for age and sex (*P* = 0.41 and *P* = 0.83, respectively). Hematologic parameters, including red blood cell (RBC) count, hemoglobin (Hb), hematocrit (Hct), and platelet (Plt) count were significantly associated with childhood ALL (*P* < 0.0001).

**Table 1:** Demographic and hematologic parameters

Parameters			Male	Female	*P*-Value
Gender	Control	Count	16 (32.0%)	34 (68.8%)	0.832
	Patient	Count	17 (34.0%)	33 (66.0%)	

		*N*	Mean ± SD	Median	IQR	*P*-Value
Age	Control	50	6.2 ± 2.8	6	5.25	0.418
	Patient	50	5.5 ± 5.4	5	4.5	
RBC count × 10^12^/L	Control	50	4.62 ± 0.55	4.67	0.79	< 0.0001
	Patient	50	3.22 ± 1.07	3.47	1.55	
Hb (g/dL)	Control	50	13.17 ± 0.88	13.2	1.33	< 0.0001
	Patient	50	8.48 ± 2.69	9.05	4.02	
HCT %	Control	50	38.29 ± 2.70	38.7	4.65	< 0.0001
	Patient	50	31.21 ± 7.46	31.79	12.35	
WBC count × 10^9^/L	Control	50	7.69 ± 1.18	7.77	1.99	0.145
	Patient	50	19.34 ± 30.30	6.3	14.58	
Platelet count × 10^9^/L	Control	50	277.50 ± 277.68	217	108	< 0.0001
	Patient	50	128.54 ± 157.54	54.5	198.25	

*Hb* Hemoglobin; *HCT* Hematocrit; *IQR* Interquartile range; *RBC* Red blood cell; *WBC* White blood cell

### HRM and sequencing results

**Fig. 1:**
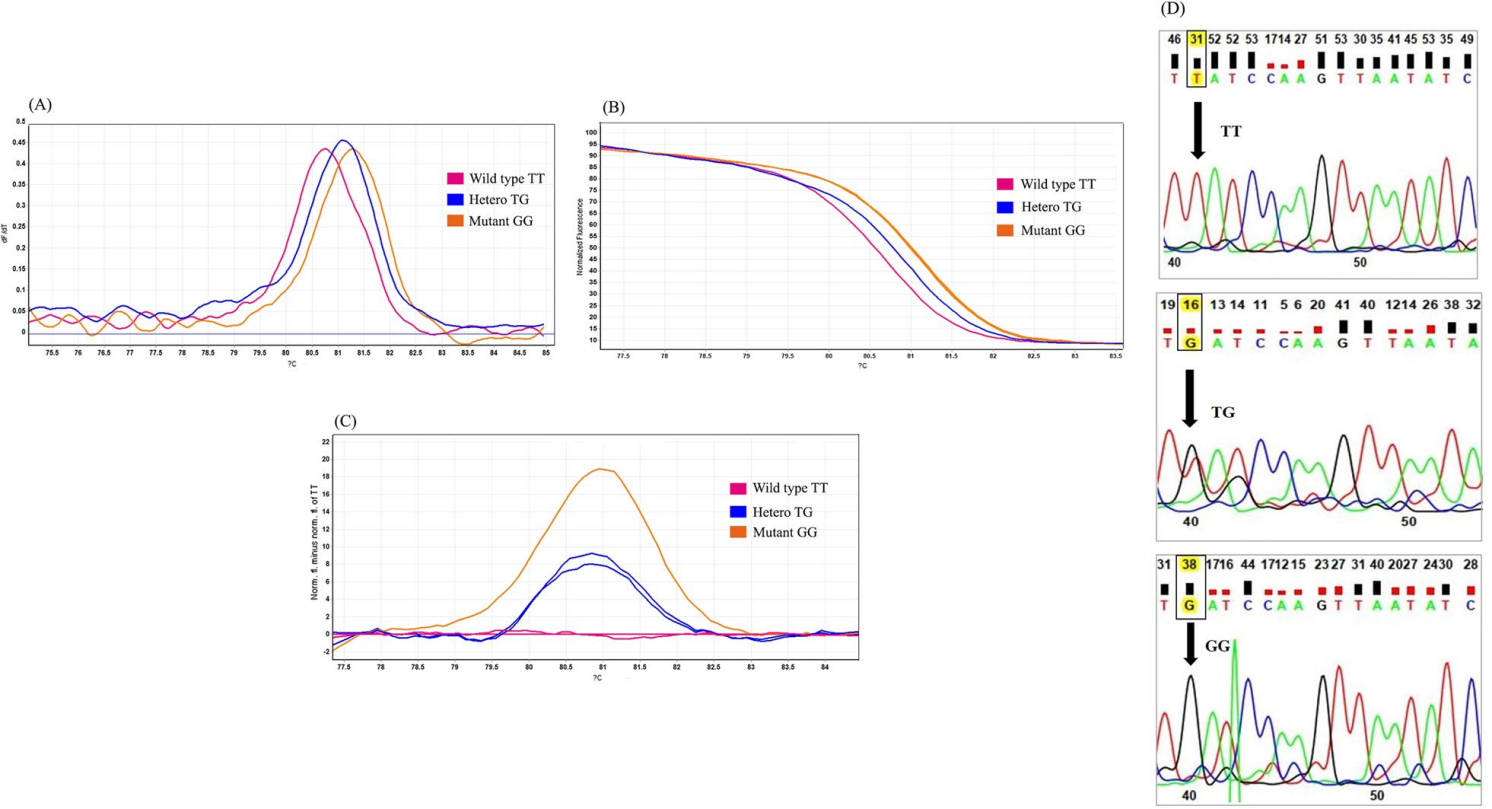
Genotyping of *IKZF1* gene polymorphism (rs4132601) by HRM analysis. **A** rs4132601 T > G Melting curve; **B** rs4132601 T > G Normalized graph; **C** rs4132601 T > G Difference graph; **D** rs4132601 T > G sequencing results

**Fig. 2:**
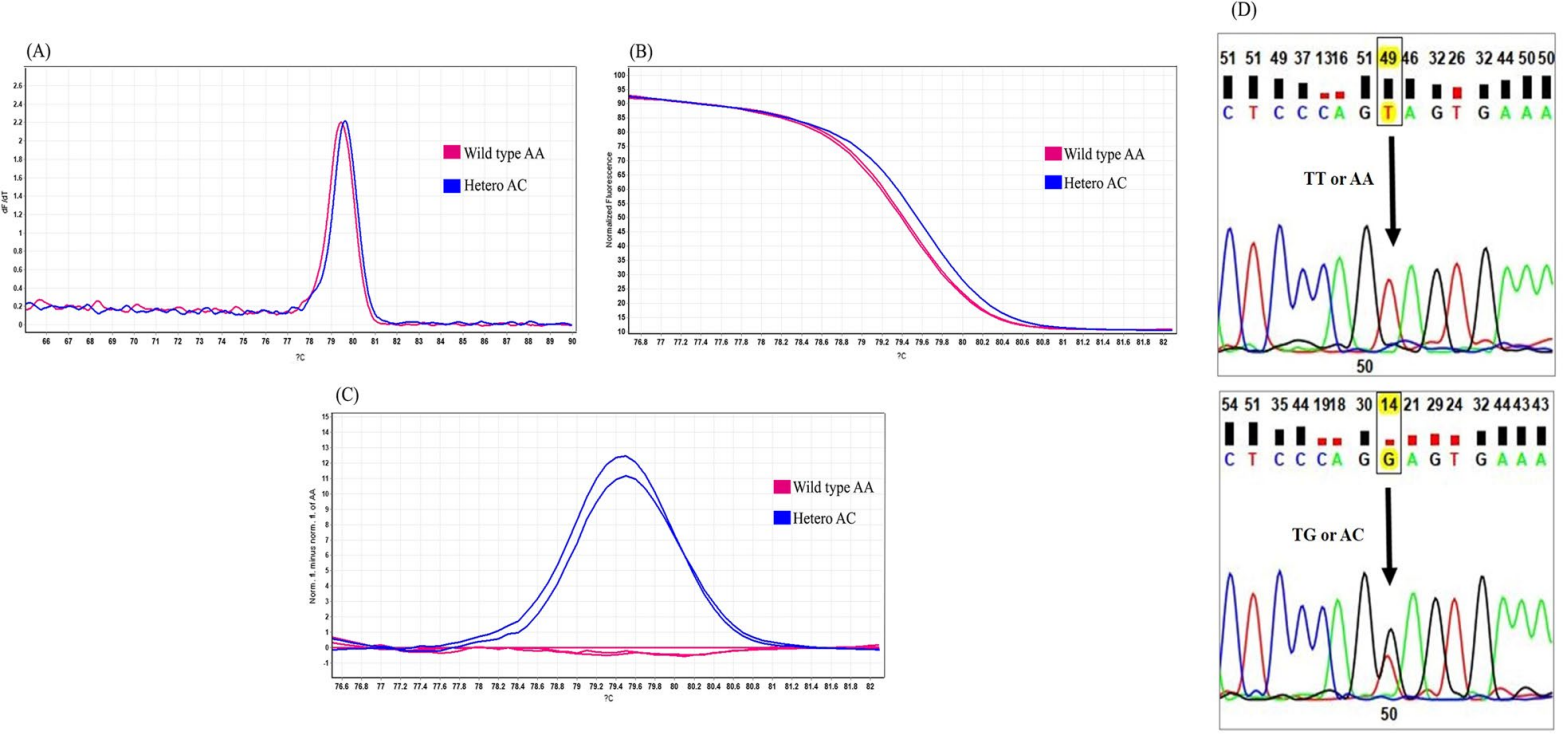
Genotyping of *CDKN2A* gene polymorphism (rs3731217) by HRM analysis. **A** rs3731217 A > C Melting curve; **B** rs3731217 A > C Normalized graph; **C** rs3731217 A > C Difference graph; **D** rs3731217 A > C sequencing results

**Fig. 3:**
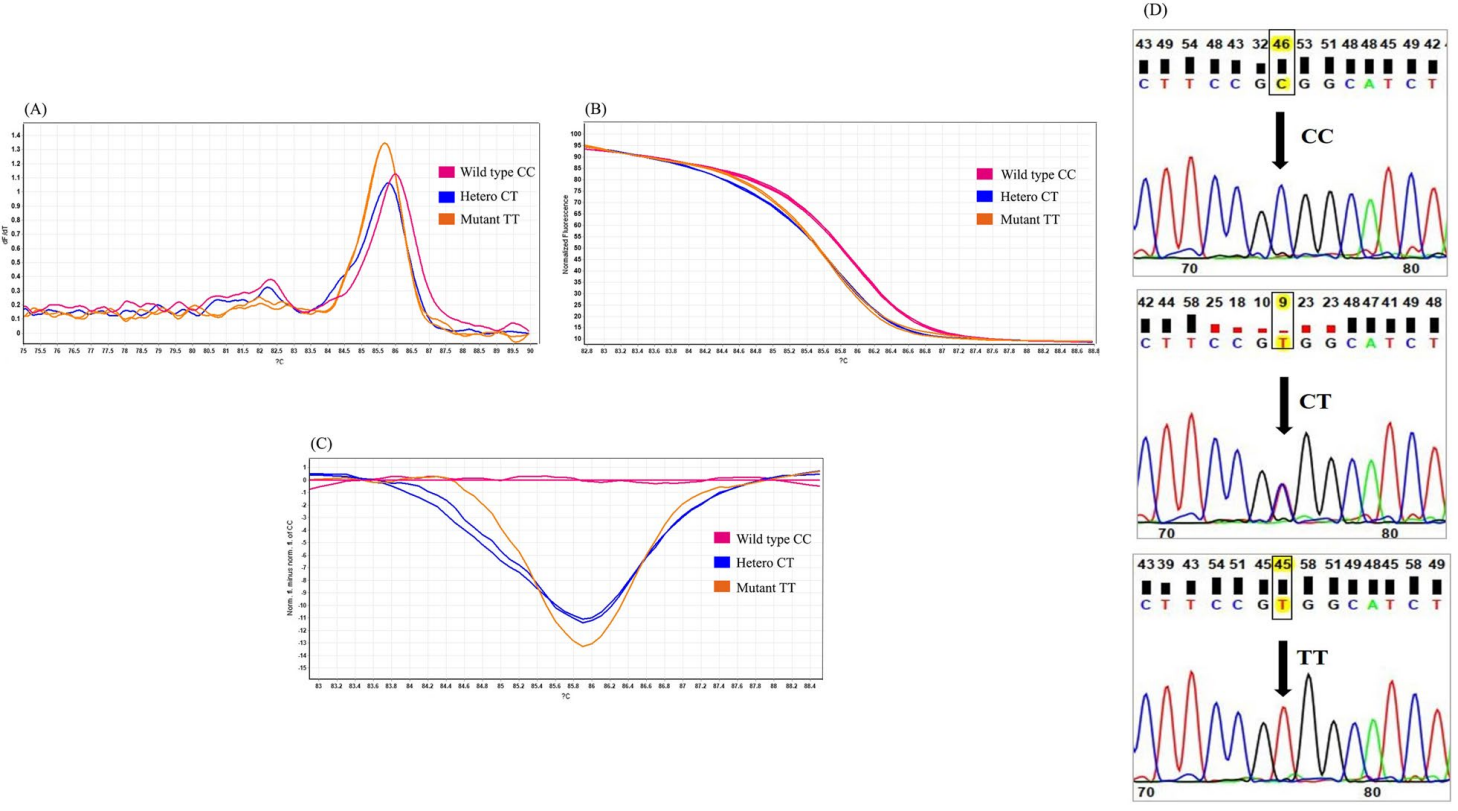
Genotyping of *CDKN2A* gene polymorphism (rs3731249) by HRM analysis. **A** rs3731249 C > T Melting curve; **B** rs3731249 C > T Normalized graph; **C**: rs3731249 C > T Difference graph; **D** rs3731249 C > T sequencing results

HRM assay was used to identify *IKZF1* (rs4132601 T > G) and *CDKN2A* (rs3731217A > C and rs3731249 C > T) gene polymorphisms. Our results revealed that the melting curves manifest different patterns, so several samples were picked to be sequenced from each group. Figures 1, 2 and 3 illustrate the sequencing results for each SNP as well as the HRM genotyping results presented in three models (melting curve, normalized graph, and difference graph).

### Genotype and allele frequency of *IKZF1* and *CDKN2A* gene polymorphisms and the association with childhood ALL

According to the findings of this study, the patients' group showed a significant deviation from Hardy–Weinberg principle (*P* < 0.05) for rs4132601 T > G (*IKZF1*) polymorphism. Nonetheless, the genotype frequencies of rs3731217 A > C and rs3731249 C > T (*CDKN2A*) polymorphisms in both case and control groups were not significantly different from the expected frequencies of Hardy–Weinberg equilibrium (*P* > 0.05). Table 2 depicts the frequency of genotypes and alleles in the patient and control groups, suggesting that both groups do not differ significantly in their genotype distribution and allele frequencies of rs4132601 (*IKZF1*) and rs3731217 (*CDKN2A*). Although we found no significant relationship between these polymorphisms and the risk of ALL in childhood, the frequency of genotype and allele of rs3731249 C > T significantly differed in the cases compared to the controls (*P* = 0.01 and *P* = 0.005 for genotype and allele frequency, respectively). Thus, our study suggested that CT and dominant model (CT + TT) genotypes and T allele of rs3731249 C > T (*CDKN2A*) variant were linked with a higher risk of childhood ALL (CT vs CC: OR = 9.56, 95% CI = 1.14–79.64, *P* = 0.037; Dominant model, CT + TT vs CC: OR = 10.76, 95% CI = 1.31–88.46, *P* = 0.004; and T allele vs C allele: OR = 11.00, 95% CI = 1.38–87.64, *P* = 0.024).

**Table 2:** Genotype and allele frequency of *IKZF1* and *CDKN2A* gene polymorphisms and the association with childhood ALL

		Group		*P-Value	OR	95% CI for OR	
		Patient	Control			Lower	Upper
*IKZF1* rs4132601 T > G							
Genotype	TT	25 (50%)	24 (48%)	–	1.0	–	–
	TG	16 (32%)	20 (40%)	0.549	0.76	0.32	1.82
	GG	9 (18%)	6 (12%)	0.543	1.44	0.44	4.66
Dominant model	TG + GG	25 (50%)	26 (52%)	0.84	0.92	0.42	2.02
Recessive model	TT + TG	41 (82%)	44 (88%)	0.4	0.62	0.20	1.90
Allele	T	66 (66%)	68 (68%)	–	1.0	–	–
	G	34 (34%)	32 (32%)	0.764	1.09	0.60	1.97
*CDKN2A* rs3731217 A > C							
Genotype	AA	48 (96%)	49 (98%)	–	1.0	–	–
	AC	2 (4%)	1 (2%)	0.565	2.04	0.17	23.26
	CC	0 (0%)	0 (0%)	–	–	–	–
Allele	A	98 (98%)	99 (99%)	–	1.0	–	–
	C	2 (2%)	1 (1%)	0.568	2.02	0.18	22.64
*CDKN2A* rs3731249 C > T							
Genotype	CC	41 (82%)	49 (98%)	–	1.0	–	–
	CT	8 (16%)	1 (2%)	**0.037**	9.56	1.14	79.64
	TT	1 (2%)	0 (0%)	–	–	–	–
Dominant model	CT + TT	9 (18%)	1 (2%)	**0.004**	10.76	1.31	88.46
Recessive model	CC + CT	49 (98%)	50 (100%)	0.24	0.00	0.00	NA
Allele	C	90 (90%)	99 (99%)	–	1.0	–	–
	T	10 (10%)	1 (1%)	**0.024**	11.00	1.38	87.64

*ALL* Acute lymphoblastic leukemia; *CI* Confidence interval; *OR* Odds ratio.

*P < 0.05 is in bold.

To explore the interaction of SNPs, we performed the compound genotype analysis for the various combinations of *IKZF1* and *CDKN2A* gene polymorphisms (in pairs and in a set of all three). As presented in Table 3, our results demonstrated that the subjects with the *CDKN2A* rs3731217 AA and *CDKN2A* rs3731249 CT genotypes exhibited significantly higher risk of developing ALL compared with those who carried the wild-type variant for both SNPs (AA/CT vs AA/CC: OR = 9.84, 95% CI = 1.18–82.14, *P* = 0.035).

**Table 3:** Association of the various compound genotypes of *IKZF1* and *CDKN2A* gene polymorphisms (in pairs and in a set of all three) with childhood ALL

		*P-Value	OR	95% CI for OR	
				Lower	Upper
*IKZF1* rs4132601*, CDKN2A* rs3731217 and *CDKN2A* rs3731249 combinations in pairs					
*IKZF1* rs4132601 */ CDKN2A* rs3731217					
	TT/AC to TT/AA	1.0	–	0.00	0.00
	TG/AA to TT/AA	0.59	0.78	0.32	1.90
	TG/AC to TT/AA	1.0	1.0	0.05	16.92
	GG/AA to TT/AA	0.5	1.5	0.46	4.87
*IKZF1* rs4132601 */ CDKN2A* rs3731249					
	TT/CT to TT/CC	0.999	–	0.00	0.00
	TT/TT to TT/CC	1.000	–	0.00	0.00
	TG/CC to TT/CC	0.555	0.76	0.31	1.86
	TG/CT to TT/CC	0.999	–	0.00	0.00
	GG/CC to TT/CC	0.901	1.09	0.27	4.28
	GG/CT to TT/CC	0.203	4.36	0.45	42.08
*CDKN2A* rs3731217 */ CDKN2A* rs3731249					
	AA/CT to AA/CC	**0.035**	9.84	1.18	82.14
	AA/TT to AA/CC	1.000	–	0.00	0.00
	AC/CC to AA/CC	0.469	2.46	0.21	28.16
*IKZF1* rs4132601*, CDKN2A* rs3731217 and *CDKN2A* rs3731249 combinations in a set of all three					
*IKZF1* rs4132601 */ CDKN2A* rs3731217 / *CDKN2A* rs3731249					
	TT/AA/CT to TT/AA/CC	0.999	–	0.00	0.00
	TT/AA/TT to TT/AA/CC	1.000	–	0.00	0.00
	TG/AA/CC to TT/AA/CC	0.599	0.78	0.31	1.95
	TG/AA/CT to TT/AA/CC	0.999	–	0.00	0.00
	GG/AA/CC to TT/AA/CC	0.849	1.14	0.29	4.50
	GG/AA/CT to TT/AA/CC	0.189	4.57	0.47	44.17
	TT/AC/CC to TT/AA/CC	1.000	–	0.00	0.00
	TG/AC/CC to TT/AA/CC	0.926	1.14	0.06	19.42

Compound wild-type genotypes were used as reference groups; *TT IKZF1* rs4132601 wild-type genotype; *AA CDKN2A* rs3731217 wild-type genotype; *CC CDKN2A* rs3731249 wild-type genotype.

*ALL* Acute lymphoblastic leukemia; *CI* Confidence interval; *OR* Odds ratio.

*P < 0.05 is in bold.

## Discussion

ALL is the most prevalent pediatric cancer globally, and genetic heterogeneity among ALL patients, resulting from differences in ethnicity and race, has a crucial role in ALL susceptibility and prognosis. Using the HRM technique, the present study evaluated the prevalence of three commonly assessed SNPs in ALL studies (rs4132601, rs3731217 and rs3731249) in the Iranian ALL patients.

Previous studies showed that two SNPs at the *CDKN2A* locus are associated with susceptibility to ALL. The first SNP, rs3731249 C > T, is located in exon 2 of the *CDKN2A* and is responsible for the Ala148Thr (Alanine to Threonine) change that reduces gene expression and affects the function of both p16^INK4A^ and p14^ARF^ proteins [15, 30]. This study found a significant association between rs3731249 genotype and allele frequency and ALL. Likewise, a report by Gutierrez-Camino et al. demonstrated that rs3731249 heightened the risk of B-ALL in the Spanish population coincided with those of this study [23]. Another study indicated this SNP to lead to a three-fold increase in ALL risk among European children [31]. Moreover, Vijayakrishnan et al. asserted that the association between rs3731249 and ALL was not limited to the distinct subgroup of B-cell ALL [32].

The other *CDKN2A* SNP that was investigated in this study is rs3731217 A > C, which is located in intron 1 of this gene [33]. The present study showed no significant association between rs3731217 A > C and susceptibility to ALL. In line with the present study's findings, Gutierrez-Camino et al. showed that rs3731217 was not associated with the ALL susceptibility [23]. According to a wide variety of comparable studies conducted on samples of Spanish [34], Polish [14], Thai [35], and Tunisian populations [30], there was no association between rs3731217 and the risk of ALL. In contrast, Sherborne et al. revealed that rs3731217 is effective on the risk of ALL, irrespective of cell lineage [33].

The last SNP that was evaluated in this study was rs4132601 T > G, which is situated in the 3^'^UTR region of the *IKZF1* gene [36]. We found an insignificant association of rs4132601 between our ALL and healthy population; thus, our study suggests that this SNP is not related to ALL susceptibility. Similarly, prior studies showed that rs4132601 T > G was not correlated with ALL [37–39]. In contrast, according to a meta-analysis performed in 2015, rs4132601 T > G heightened the risk of ALL and the most significant degree of association was observed among European and Spanish populations, though not in the Asian one [19]. The meta-analysis conducted by Dai et al. indicated that rs4132601 in the *IKZF1* gene was likely to contribute to the incidence of ALL in European populations [40]. Likewise, Bahari et al. found that GG and TG genotype and G allele of rs4132601 T > G can be considered as ALL risk factors [16].

To the best of our knowledge, this is the first study to investigate in detail the association between the developing pediatric ALL and the presence of various combinations of *IKZF1* (rs4132601) and *CDKN2A* (rs3731217 and rs3731249) gene polymorphisms (in pairs and in a set of all three). The combination of *CDKN2A* rs3731217 AA and *CDKN2A* rs3731249 CT genotypes significantly increased the risk of developing childhood ALL.

Our study has some limitations. The main limitation of this study is the small sample size. In fact, all available samples were collected over a period of almost two years, but we could not achieve a larger sample size. Regarding the causes of this issue, in addition to the time constraint, the study population was leukemic children undergoing chemotherapy and so many parents refused to participate in this study. Moreover, the results of studies about ALL's SNPs in various ethnicities are inconsistent. Therefore, we suggest that further studies be performed in various populations with larger sample size to validate our findings. Another limitation that prevented us from investigating the effect of these SNPs on treatment response prediction or risk of relapse was the time constraint. Examining the association of these SNPs with the patient's response to treatment and the risk of recurrence as well as analysis of other genes involved in ALL pathogenesis, will give significant insight into this disease.

In conclusion, we identified rs3731249 C > T, a *CDKN2A* missense SNP, as an essential predictor of ALL susceptibility. We hope that our results might serve as a useful ALL diagnostic marker. Furthermore, *CDKN2A* rs3731249 polymorphism produces a dysfunctional protein; and inhibition of this protein with targeted therapy modalities can be recommended for disease treatment.

## Data Availability

The datasets used and/or analyzed during the current study are not publicly available due to the possibility of compromising the privacy of individuals. According to the written approval forms accepted by the Ethics Committee of the Kerman University of Medical Sciences (KMU), the data will only be accessible to researchers within the project. The data would be available from the corresponding author on reasonable request.
